# Pancreatic pseudocyst drainage in children by image-guided cystogastrostomy and stent insertion

**DOI:** 10.1007/s00247-019-04471-9

**Published:** 2019-07-24

**Authors:** Premal A. Patel, Craig Gibson, Kishore S. Minhas, Sam Stuart, Paolo De Coppi, Derek J. Roebuck

**Affiliations:** 1 Interventional Radiology, Radiology Department, Great Ormond Street Hospital, Great Ormond Street, London, WC1N 3JH UK; 2 Wellcome/EPSRC Centre for Interventional & Surgical Sciences, University College London, London, UK; 3 Department of Medical Imaging, Perth Children’s Hospital, Perth, Australia; 4 Department of Specialist Neonatal and Paediatric Surgery, Great Ormond Street Hospital, London, UK; 5 Stem Cells and Regenerative Medicine Section, UCL Institute of Child Health and Great Ormond Street Hospital, University College London, London, UK; 6 Discipline of Paediatrics, Medical School, University of Western Australia, Perth, Australia

**Keywords:** Adolescents, Children, Cystogastric stent, Interventional radiology, Pancreas, Pseudocyst

## Abstract

**Background:**

Endoscopic ultrasound is seldom available at paediatric centres; therefore drainage of pancreatic pseudocysts in children has traditionally been achieved by surgery.

**Objective:**

This study assessed the feasibility and safety of performing image-guided internal drainage of pancreatic pseudocysts with a flanged self-expanding covered nitinol pancreatic pseudocyst drainage stent.

**Materials and methods:**

We conducted a retrospective case note review of children undergoing image-guided cystogastrostomy at two paediatric hospitals. Percutaneous access to the stomach was achieved via an existing gastrostomy tract or image-guided formation of a new tract. Under combined ultrasound, fluoroscopic or cone-beam CT guidance the pancreatic pseudocysts were punctured through the posterior wall of the stomach. A self-expanding covered nitinol stent was deployed to create a cystogastrostomy opening.

**Results:**

Image-guided cystogastrostomy was performed in 6 children (4 male; median age 6 years, range 46 months to 15 years; median weight 18 kg, range 13.8–47 kg). Two children had prior failed attempts at surgical or endoscopic drainage. Median maximum cyst diameter was 11.5 cm (range 4.7–15.5 cm) pre-procedure. Technical success was 100%. There were no complications. There was complete pseudocyst resolution in five children and a small (2.1-cm) residual pseudocyst in one. Pseudocyst-related symptoms resolved in all children.

**Conclusion:**

Pancreatic pseudocyst drainage can be successfully performed in children by image-guided placement of a cystogastrostomy stent. In this cohort of six children there were no complications.

## Introduction

Pancreatic pseudocysts are collections of pancreatic secretions that are lined by fibrous tissues and might contain necrotic debris or blood. Pseudocyst formation is a well-recognised complication of acute pancreatitis, chronic pancreatitis and pancreatic trauma. In children the most common cause is pancreatic trauma [1]. Pseudocysts are usually located within or adjacent to the pancreas itself in the lesser sac [2]. Most pancreatic pseudocysts in children resolve spontaneously following conservative management of bowel rest, supportive nutrition and analgesia [3]. Pseudocysts that become symptomatic, persist for 6 weeks or more, or continue to increase in size, especially beyond 6 cm, usually require therapeutic intervention [3, 4]. For collections secondary to trauma, intervention is considered if the symptoms or collection persist for a week after withholding enteral nutrition [5]. Pancreatic pseudocysts can be managed by surgical, percutaneous or endoscopic drainage [1]. In children, drainage of pseudocysts has traditionally been accomplished by surgical intervention and more recently by interventional endoscopy assisted by endoscopic ultrasound. However interventional endoscopy and endoscopic ultrasound are not always available in paediatric centres. Therefore the assistance of paediatric interventional radiology is sometimes sought in managing these children. This study assessed the feasibility and safety of performing image-guided internal drainage of pancreatic pseudocysts with a flanged self-expanding covered nitinol pancreatic pseudocyst drainage stent.

## Materials and methods

This two-centre retrospective study was exempted from institutional review board approval. We identified children by conducting searches of prospectively maintained interventional radiology procedures databases. We retrospectively reviewed the electronic medical records of all children between the ages of 0 and 18 years undergoing percutaneous pancreatic pseudocyst drainage using a flanged self-expanding covered nitinol pancreatic pseudocyst drainage stent at one of two large tertiary paediatric institutions between December 2013 and December 2017. There were no exclusion criteria. We assessed procedural indications, procedural and relevant prior imaging, technical details, and clinical and imaging follow-up. Three of the study investigators (P.A.P., C.G. and D.R. who have an average of 10 years of post-fellowship practice experience [range 4–23 years]) reviewed the data.

### Study definitions and criteria

The primary endpoint of technical success was defined as successful placement of a cystogastric stent. The secondary endpoints were complications, categorised using Society of Interventional Radiology criteria [6], and outcome, defined by residual pseudocyst size at imaging and symptoms at clinical follow-up. We used review of the medical records up to post-procedure discharge from hospital to identify and categorise delayed complications.

We analysed demographics, technical details and complications using descriptive statistics.

### Technique

**Fig. 1:**
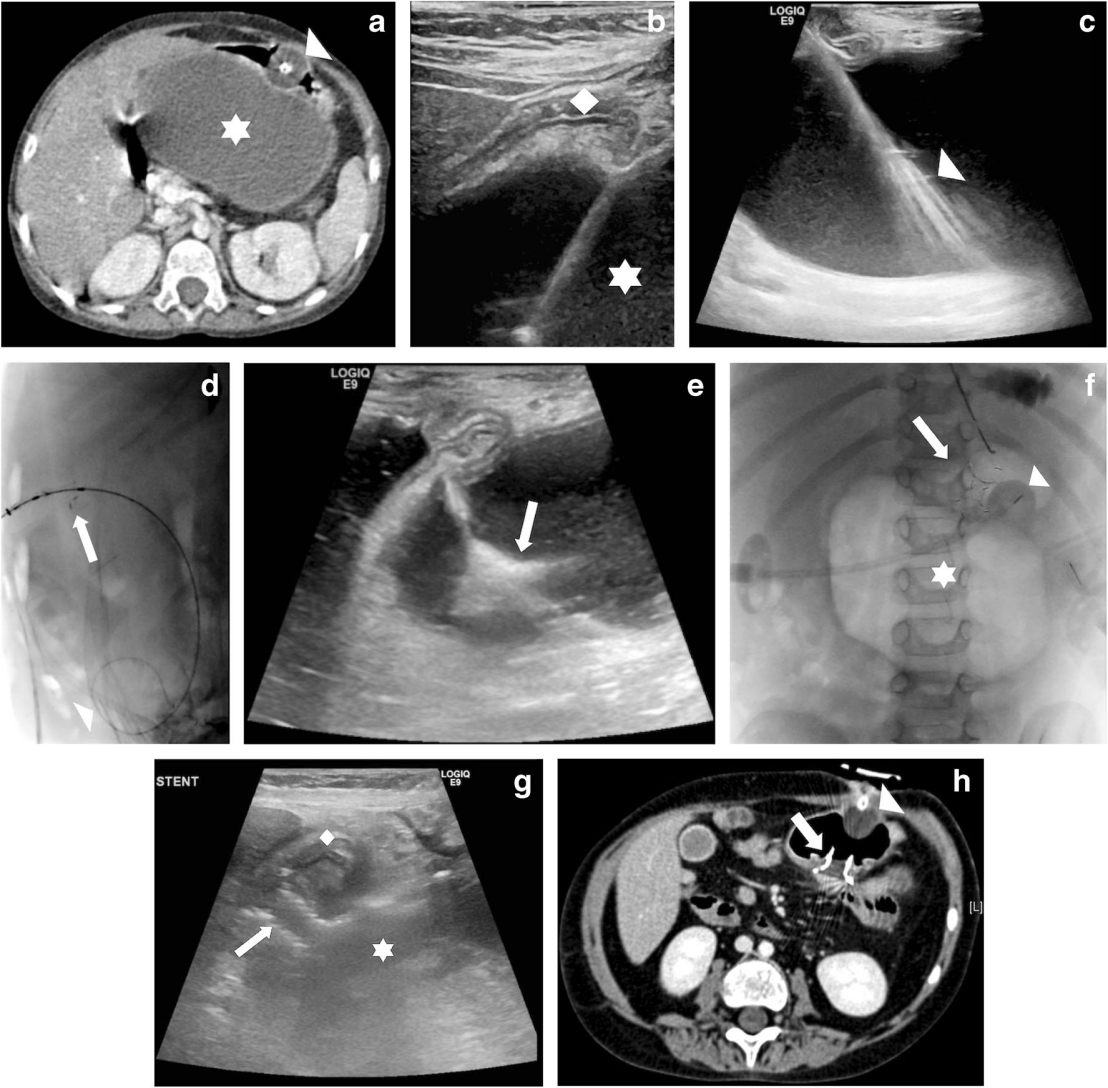
Pancreatic pseudocyst in a 6-year-old girl (Table 1, patient 1). **a** Contrast-enhanced axial CT image demonstrates a large pancreatic pseudocyst (*star*) and compressed stomach with balloon-retained gastrostomy (*arrowhead*). **b** Sagittal US image demonstrates puncture of the pseudocyst (s*tar*) through the stomach (*diamond*). **c** Sagittal US image demonstrates guidewire insertion and direction to deep component of pseudocyst, where it was coiled (*arrowhead*). **d** Lateral fluoroscopic image demonstrates coiled wire (*arrowhead*) and the start of stent deployment with flaring of distal end (*arrow*). **e** Sagittal US image confirms the distal flange flaring in the pseudocyst (*arrow*). **f** Anteroposterior fluoroscopic image demonstrates final stent position *(arrow)*. There is gas in the pseudocyst (*star*). A balloon-retained gastrostomy is in situ *(arrowhead).***g** Sagittal US image day 1 post-procedure demonstrates stent position (*arrow*). The stomach *(diamond)* is empty and the pseudocyst (*star*) is smaller than before the procedure. **h** Contrast-enhanced axial CT image 1 month after stent insertion demonstrates the stent (*arrow*) and resolution of the pseudocyst. A balloon-retained gastrostomy is present (*arrowhead*)

Children were referred for radiologic drainage of large, non-infected, symptomatic or complicated pancreatic pseudocysts. The presence of a pseudocyst was confirmed by imaging studies. Pre-procedure imaging included CT or MRI and ultrasound (US) to provide information on size, location and number of cysts (Fig. 1). As at other institutions [7], children with symptomatic pseudocysts are referred for drainage but the integrity of the pancreatic duct is not routinely imaged by endoscopic retrograde cholangiopancreatography or MR cholangiopancreatography. The decision regarding drainage approach was made at multidisciplinary team meetings, where children were assigned for either surgical or radiologic drainage. At our institutions the availability of interventional endoscopy and endoscopic ultrasound is limited.

All procedures were performed under general anaesthesia by one of four paediatric interventional radiologists (P.A.P., C.G., S.S., D.J.R.) with between 4 and 19 years experience. Prophylactic antibiotics were administered according to interventional radiologist preference. Epigastric US visualisation of the pancreatic pseudocyst and access planning was performed using either a Logiq E9 (GE Healthcare, Waukesha, WI) or Acuson S3000 (Siemens Healthcare, Munich, Germany). Percutaneous access to the stomach was performed via an existing gastrostomy tract if one existed. Otherwise, following air or saline insufflation via a nasogastric tube, the stomach was punctured under combination US and bi-plane (anteroposterior and lateral projections) fluoroscopy using an Artis Zee or Axiom Artis dBC (Siemens). To facilitate stomach insufflation, glucagon (0.03 mg/kg up to a maximum dose of 1 mg) was administered. Two or three gastropexy anchors (Cope Suture Anchor Set, Paediatric Configuration; Cook Medical, Bloomington, IN) were delivered according to radiologist preference. Combined US, fluoroscopic or cone-beam CT guidance was used to access the pancreatic pseudocyst with an 18-gauge trocar needle through the posterior wall of the stomach (Fig. 1). Iohexol (Omnipaque 240; GE Healthcare) was injected to confirm intra-pseudocyst position. A 0.035-in. stiff guidewire (Amplatz Extra-Stiff Wire Guide or Amplatz Super-Stiff Wire Guide; Cook Medical) was advanced into the pseudocyst (Fig. 1). Over the wire, the cystogastric tract was dilated using a 5-mm or 8-mm angioplasty balloon. In some cases, depending on radiologist preference, an 11-French (Fr) or 12-Fr sheath was advanced into the pseudocyst over the wire. A self-expanding covered nitinol stent designed for pancreatic pseudocyst drainage (Niti-S Nagi; Taewoong, Gyeonggi-do, South Korea) was deployed (Fig. 1). The stent size was chosen based on pre- and intra-procedural assessment of distance from gastric lumen to pseudocyst lumen. The stent was deployed under US and fluoroscopic guidance. After deployment of the distal flange, the delivery sheath was gently and slowly pulled so the distal flange was aligned with the internal cavity of the cyst. The rest of the stent was then deployed. Through the stent, the pseudocyst was aspirated. If there was a pre-existing gastrostomy, this was replaced. If there was no pre-existing gastrostomy, the gastrostomy tract formed to perform the drainage was allowed to close. If used, gastropexy sutures were cut approximately 2 weeks after the procedure.

## Results

### Patient population

Image-guided cystogastrostomy was performed in six children (four male), ages 46 months to 15 years (median age 6 years), with weight between 13.8 kg and 47 kg (median weight 18 kg; Table 1). The cause of the pseudocyst was chronic pancreatitis in two children (associated with hypermobility-type Ehlers–Danlos syndrome in one child), acute pancreatitis in two children (10 weeks following heart transplant in one and following commencement of asparaginase for acute lymphoblastic leukaemia in another), recurrent pancreatitis in one child secondary to a serine protease 1 *(PRSS1)* mutation, and blunt-traumatic transection of the pancreas in one child.

**Table 1:** Summary of all children treated by image-guided pancreatic pseudocyst drain using a covered nitinol stent

Patient	Age, gender	Weight (kg)	Cause of pseudocyst	Pancreatic pseudocyst maximum diameter (cm)	Previous attempted drainage	Stent size (width x length, mm)	Image guidance used	Maximum diameter of pseudocyst at imaging follow-up (cm)	Length of imaging follow-up (days)
1	6 y 11 mo, f	16.4	Chronic pancreatitis	13.1	None	10×10	US & fluoroscopy	0	67
2	15 y 7 mo, m	47.0	Acute pancreatitis	8.7	None	10×20	US, fluoroscopy & cone-beam CT	2.1	96
3	6 y 4 mo, m	19.6	Chronic pancreatitis	4.7	Surgical	10×20	US, fluoroscopy & cone-beam CT	0	583
4	4 y 2 mo, m	16.1	Traumatic transection of pancreas	10.0	Endoscopic	10×20	US & fluoroscopy	0	50
5	13 y 5 mo, f	36.5	Acute pancreatitis	15.5	None	10×30	US & fluoroscopy	0	216
6	3 y 10 mo, m	13.8	Hereditary pancreatitis	13.0	None	10×30	US & fluoroscopy	0	98

*CT* computed tomography, *f* female, *m* male, *mo* months, *US* ultrasonography, *y* years

Two children had a prior failed attempt at surgical or endoscopic drainage. In the child who underwent surgery, 10 weeks prior to the image-guided pseudocyst drainage procedure there was re-accumulation. The other child had an attempted endoscopic drainage 5 weeks prior to the image-guided pseudocyst drainage procedure but the attempt was abandoned because a puncture of the pseudocyst was deemed impossible. CT (4), MR cholangiopancreatography (1) and both CT and MR cholangiopancreatography (1) performed a median of 8 days (range 2–36 days) before the procedure showed median maximum cyst diameter of 11.5 cm (range 4.7–15.5 cm).

### Technique

Two children (patient 1 and patient 3) had existing gastrostomies, which were used for initial access to the stomach. A new puncture was made in the other four children following deployment of gastropexy sutures in two children. Five children received prophylactic broad-spectrum antibiotics at induction of general anaesthesia. One of the two children with a pre-existing gastrostomy and all children in whom a new stomach puncture was required received antibiotics.

### Outcomes

A self-expanding covered nitinol pancreatic pseudocyst drainage stent (Niti-S Nagi) was successfully placed under image guidance in all children. There were no immediate or delayed complications. Children were discharged a median of 11 days (range 5–70 days) post-procedure. The longest post-procedure inpatient stay of 70 days was the child being treated for acute lymphoblastic leukaemia. Pseudocysts completely resolved in five of the six children. Follow-up cross-sectional imaging by CT (*n*=5) or MR cholangiopancreatography (*n*=1) was performed at a median of 54 days (range 12–98 days). Four children had further imaging follow-up by US (*n*=2) or CT (*n*=2). Initial imaging follow-up showed complete pseudocyst resolution in four children (Fig. 1). There were residual pseudocysts in two children measuring 2.1 cm and 3.5 cm. The 3.5-cm residual collection, seen on early CT follow-up 12 days post-procedure, had resolved before CT 216 days post-procedure (Table 1, Patient 5). The child with a 2.1-cm residual collection did not undergo further imaging follow-up (Table 1, Patient 2). There was no re-accumulation at long-term imaging performed at a median of 209 days (range 67–583 days). At last clinical follow-up, a median of 723 days (range 55–967 days) after the procedure, pseudocyst-related symptoms had resolved in all children. Two stents were removed endoscopically 55 days and 237 days after insertion, and one was noted to be absent at CT 96 days post-procedure (Patient 2) and was presumed to have passed naturally per rectum. Three stents remained in situ.

## Discussion

Pancreatic pseudocysts are a rare but potentially troublesome problem in children. They can occur as the result of pancreatic insult with ductal disruption, often secondary to trauma. Pseudocyst drainage is warranted when attempts at conservative management fail. Current approaches to draining pancreatic pseudocysts include open or laparoscopic surgery, endoscopic drainage, and image-guided percutaneous internal or external drainage.

Surgical drainage has been the traditional method [8]. This is traditionally performed by an open approach, and more recently by laparoscopic techniques that attempt to replicate their open equivalents [9]. Surgical techniques include formation of a cystogastrostomy if the pseudocyst is adherent to the posterior wall of the stomach, or otherwise formation of a Roux-en-Y cystojejunostomy [9]. Surgery is particularly useful in people with unfavourable anatomy [9].

Endoscopic drainage of pseudocysts was introduced in the mid-1980s and is reported to be safe and effective. It has since become the primary therapeutic modality for drainage of pseudocysts [1, 3]. The use of endoscopic ultrasound has evolved more recently [10, 11]. Endoscopic ultrasound can help in finding the optimal site for puncture of the pseudocyst and subsequent stent placement by assessing wall thickness and the distance from stomach or duodenum to pseudocyst, and by identifying major vascular structures [3, 10, 11]. However endoscopic ultrasound has been limited mostly to diagnostic use in children, with only a few case reports describing its therapeutic role in pseudocysts [3].

Endoscopic drainage can be performed from transpapillary or transmural approaches. Endoscopic transmural drainage is usually performed with endoscopic US guidance. Endoscopic and endoscopic US-guided transmural drainage require direct apposition with visible bulging of the gastric or duodenal wall at the site of the pseudocyst and a cyst wall thickness less than 1 cm [1, 9, 12], and drainage is usually achieved by placement of one or two plastic stents or a metal stent.

Percutaneous drainage can be performed with US or CT guidance and can be achieved by a retroperitoneal or transperitoneal route [9]. Percutaneous drainage is now reserved for unstable patients, immature cysts, and patients with infected pancreatic pseudocysts [13]. It is infrequently performed because of the risk of creating a cystocutaneous fistula.

Percutaneous cystogastrostomy formation has also been reported in adults [7, 14, 15]. This has been done using double pigtail catheters with US and fluoroscopic guidance [7, 14]. Initially this was a two-stage procedure involving placement of a stent between the stomach and pseudocyst after 7 days [15], but more recently single-step procedures have been reported [7]. In adult series of up to 12 patients percutaneous cystogastrostomy has been shown to have a high success rate with good short-term outcomes [7]. This technique avoids the incisions of up to 2 cm that are made in the gastric wall with a knife during endoscopic drainage and can cause bleeding or perforation of the peritoneum [7].

There is debate regarding the approaches to treatment of pancreatic pseudocysts even in adults, in whom they occur much more frequently than in children. A systematic review comparing the outcomes of endoscopic, percutaneous and surgical pancreatic pseudocyst drainage included results from 10 comparative studies, of which three were randomised controlled trials [9]. It found no consensus about the best management approach [9]. Endoscopic US-guided drainage appeared to be advantageous for pancreatic pseudocysts located adjacent to the stomach or duodenum. Surgical cystojejunostomy or percutaneous drainage can be considered for people with unfavourable anatomy [9]. There have been reports of the successful use of all of these approaches in children but, as in adults, there is no consensus regarding best treatment. Our series of six children, in which technical success was 100%, demonstrates that cystogastrostomy using interventional radiology techniques is feasible in children and therefore might be a useful alternative to surgery or interventional endoscopic ultrasound. The recurrence rate after surgery has been reported to be as high as 10% [1]. This series included one child who had re-accumulation after a previous attempt at surgical drainage, but there were no pancreatic pseudocyst re-accumulations in our patients after image-guided cystogastric stent insertion.

It has been recommended that all cystoenterostomies be stented to avoid recurrence [10]. Often two double-pigtail plastic stents are placed to maintain patency, allow complete resolution of the pseudocyst and minimise the effect of spontaneous stent migration [10], but these often need replacement because of dysfunction and migration [13]. Further, endoscopic placement of multiple plastic stents can be technically difficult, so the use of a single completely covered self-expanding metallic stent has been proposed as an alternative [13]. These devices allow a single-step creation of a large-diameter fistula [13]. In a number of studies, including a recent meta-analysis, of metal versus plastic stents for drainage of pancreatic fluid collections, the use of metal stents was found to be associated with improved clinical success, fewer adverse events and reduced bleeding compared to plastic stents [16, 17]. However stent migration occurs in up to 15% of patients [13] and bleeding requiring embolisation, stents becoming buried under gastric mucosa, and a biliary stricture related to mechanical compression from a stent have all been reported [18]. Additionally, even in adults, there is a paucity of long-term safety data.

The self-expanding covered nitinol pancreatic pseudocyst drainage stent used in this case series (Niti-S Nagi), although designed for endoscopic placement, can be placed using image guidance and a transgastric approach. The advantage of this device is that the large (20-mm) acute-angled flared ends anchor the stent in the stomach and pseudocyst and help prevent migration [13]. It should be noted that according to information from the manufacturer, a stent might require 1–3 days to expand fully and a soft diet is suggested after insertion. This stent has been reported to have been used for endoscopic US-guided drainage in 21 children (mean age 14.9 years) with technical and clinical success rates of 100% and 95%, and no major complications [19]. However stent migration was noted in 1 child [19], as in our series, and occurred in 4 of 21 adults in another series [20]. Another advantage of this device is the ease of deployment. Other image-guided cystogastrostomy series have used biliary endoscopic stents designed to treat calculi (overall length 10 cm) and have reported that they are too long and therefore difficult to deploy when the stomach lumen is reduced by the mass effect of the cyst [7]. The stents used in this study are available in lengths of 10–30 mm and therefore do not cause this problem. Another advantage of the stent is that it is of sufficient size to allow endoscopic necrosectomy should this become required.

It is recommended that flanged self-expanding covered nitinol pancreatic pseudocyst drainage stents be removed after the confirmation of complete resolution of the pseudocyst [20]. The stents should be removed endoscopically by grasping the retrieval string with forceps and collapsing the proximal end of the stent with a snare and then carefully pulling. In a study in which an attempt to remove a flanged self-expanding covered nitinol pancreatic pseudocyst drainage stent was made in 16 people, all stents were easily removed with no complications 12 months after placement [20].

Complications of percutaneous image-guided drainage appear to be uncommon. In a study of 12 people who underwent image-guided cystogastrostomy, there was post-procedural sepsis in two people with ongoing pancreatitis and only partial drainage in another [7]. In our series of six children there were no complications as a result of intervention. Reported complications of endoscopic drainage of pancreatic pseudocyst include bleeding, intestinal perforation, infection, leakage, stent migration and pseudocyst recurrence [1]. Endoscopic drainage has fewer complications than surgical drainage, which has been shown in adults to have a morbidity of up to 35% and mortality of up to 10% [3, 21].

In this series a pre-existing gastrostomy tract was used to access the stomach in two children. In such cases, the stomach is already adherent to the anterior abdominal wall and a new stomach puncture does not have to be performed. Four children in this study required a gastrostomy to be formed as part of the drainage procedure. Complications of gastrostomy formation by interventional radiology are reported to be 0–5% including peritonitis in up to 3% of cases, other infective complications such as subcutaneous abscess in 2% and septicaemia in 1%, and bowel transgression in 0.2% of cases [22]. Insufflation of air to facilitate stomach puncture can obscure subsequent pseudocyst visualisation by US and should be avoided if possible. If necessary, insufflation of the stomach with saline is a better alternative that allows for continued sonographic visualisation of the pseudocyst. Gastropexy sutures were not used in two of the four children in which there was a new stomach puncture. These children did not develop complications, suggesting the hole created in the anterior surface by the up to 12-Fr dilator closed without significant peritoneal soiling.

Pancreatico-cutaneous fistula can occur after drainage of pseudocysts and can be very difficult to treat. The risk of fistula formation might be reduced by performing a single-step procedure because the gastrostomy created is allowed to heal [7]. Avoiding the use of gastropexy devices might further reduce this risk. External drainage is usually avoided in adults because of a high incidence of pancreatico-cutaneous fistula formation, although this risk is lower in children [23].

This retrospective study has some limitations that must be acknowledged. The number of children included is small; however this reflects the rarity of the condition. Also, the precise technique including the use of prophylactic antibiotics, the tract dilation method and the use of gastropexy sutures varied slightly among patients. The generalisability of the technique to other paediatric hospitals is unknown. Endoscopic US-guided drainage has become common in adults but its application in children is lagging [3]. This is presumably because of the lack of experience in paediatric hospitals related to the rarity of this condition [3]. In small children, endoscopic US drainage can be technically difficult because of the large size of the US endoscope [19]. The requirement for specialised equipment might also limit the use of endoscopic US-guided drainage in children. Adopting a radiologic approach to drainage might have similar drawbacks. Training in low-volume, high-complexity procedures is a known problem in paediatric interventional radiology [24]. Paediatric interventional radiology procedures involve a set of core skills, however, involving access to the appropriate site under image guidance (often using a needle or catheter and guidewire) followed by some type of intervention [24], so most paediatric interventional radiologists could undertake pancreatic pseudocyst drainage.

## Conclusion

This series demonstrates that internal pancreatic pseudocyst drainage can be successfully performed by image-guided placement of a cystogastrostomy stent. This can help avoid the need for invasive surgical procedures, especially at institutions without access to interventional endoscopy and endoscopic ultrasound. In this small cohort of six children there were no complications.
